# Impact of Crisis Line Volunteering on Mental Wellbeing and the Associated Factors: A Systematic Review

**DOI:** 10.3390/ijerph17051641

**Published:** 2020-03-03

**Authors:** Renate Willems, Constance Drossaert, Patricia Vuijk, Ernst Bohlmeijer

**Affiliations:** 1 Research Center Innovations in Care; Rotterdam University of Applied Science, 3015EK Rotterdam, The Netherlands; p.vuijk@hr.nl; 2 Department of Psychology, Health and Technology, University of Twente, 7522 NB Enschede, The Netherlands; c.h.c.drossaert@utwente.nl (C.D.); e.t.bohlmeijer@utwente.nl (E.B.)

**Keywords:** crisis line, volunteer, mental wellbeing, influencing factors, systematic review

## Abstract

Crisis line services, operated by volunteers, have been proven to be effective in decreasing psychological pain and preventing suicidality. Although working at the crisis line may be rewarding, for some the confrontation with highly complex topics (i.e., suicidality, abuse, and loneliness) in combination with inappropriate calls (i.e., sexually abusive calls), may lead to distress or vicarious trauma. The aim of this paper is to systematically review the studies that have examined mental wellbeing of crisis line volunteers and the factors associated with it. Thirteen published empirical studies on the topic were found. These showed that crisis line volunteers are at increased risk of declined mental wellbeing. However, a wide range of operationalizations were used and most studies did not use validated instruments. On the other hand, studies showed that many volunteers experience satisfaction and gratification from their work. This review gives insight into some of the work-related, organization-related, and volunteer-related factors that may be associated with the decrease of mental wellbeing. More high quality, comprehensive, and quantitative research using validated instruments is urgently needed to assess the impact of the work on mental wellbeing and the relative impact of influencing factors.

## 1. Introduction

Crisis line services, operated by volunteers, can be considered as an important addition to the existing formal care for people who cannot or do not want to use formal care. Crisis line services offer immediate emotional support by telephone, chat or email in case of personal crisis [1]. In Europe, there are more than 21,000 trained crisis line volunteers, who are available day and night to provide emotional support to vulnerable people in need of immediate help. These volunteers conduct over five million telephone calls and 130,000 chat and email conversations each year [1]. Crisis line services have been shown to be effective in decreasing feelings of hopelessness and psychological pain [2,3] and even in preventing suicidality [3].

Volunteering at the crisis line requires great mental flexibility, because volunteers are confronted with intense suffering and continuously need to switch between a wide range of intrusive and complex topics, such as loneliness, insomnia, suicidal thoughts, and abuse experiences [1,4]. In addition, volunteers have to deal with other call complications and/or inappropriateness, such as frequent callers (calling several times a day, with the same story) [5,6] and sexual abusive calls (in which the caller’s goal is to gain sexually gratification from the call) [7,8]. It is important that attention is paid to the impact of volunteering on the mental wellbeing of crisis line volunteers, because a decline in mental wellbeing is associated with poorer quality and safety of care, higher absenteeism, and higher turnover rates [9]. In addition, insight into the factors that are associated with mental wellbeing is needed in order to develop strategies to improve mental wellbeing in crisis line volunteers. 

Mental wellbeing and the factors affecting it have been extensively studied in professional health care workers, but research among crisis line volunteers is scare. Studies in nurses, social workers, and psychologists have identified various positive effects of caring for others, such as compassion satisfaction (the pleasure experienced by caring for others [10]) and job satisfaction [11,12]. However, negative effects have also been reported, for instance elevated symptoms of anxiety and depression [13,14], symptoms of burnout [15,16], secondary traumatic stress (experience of emotional disruption due to helping a traumatized person) [17,18], and compassion fatigue (the physical, emotional, and psychological effects of exposure to traumatic stories or events) [19,20,21].

The factors associated with mental wellbeing were also extensively studied in professional caregivers. They can be divided into three categories [10]: (a) The nature of the work; (b) the organization; and (c) characteristics of the care providers themselves. Factors related to the nature of the work that have been associated with mental wellbeing of nurses or other professional caregivers are, for example, the severity of the disease (e.g., caring for patients who are dying) [20] and having to deal with patients who are hostile or suicidal [14,15,16]. Organizational factors associated with a decrease of caregivers’ wellbeing are, for example, lack of support by managers [11,13,15,19], lack of respect and recognition [11,12], insufficient training [14], and lack of autonomy [12]. Finally, characteristics of the caregiver that have been related to decreased wellbeing include maladaptive and emotion focused coping styles [17,19], and feeling too preoccupied with patients [19,21]. All these factors may apply to crisis line volunteering as well.

Despite this substantial body of knowledge on the mental wellbeing of professional caregivers, much less research had been conducted on the impact of caregiving in crisis line volunteers. In a recent systematic review, Kitchingman et al. [22] analyzed seven studies investigating whether telephone crisis support workers experience elevated symptoms of psychological distress. The results revealed that telephone crisis support workers are experiencing stress, burnout, vicarious trauma, and psychiatric disorders. Despite the usefulness of this first review, it has two limitations. First, the review did not examine potential positive effects of volunteering. Insight into the positive experiences, motivations, and satisfaction is, however, important because positive feelings may compensate for any distress and may help to understand why people continue volunteering. Second, the review did not focus on potential factors associated with distress and mental wellbeing in crisis line volunteers. Yet insight into these factors is important for informed development and implementation of organizational or personalized interventions aimed at increasing the mental wellbeing of volunteers.

The aim of this paper is therefore to conduct a systematic review of studies that have examined positive and negative mental wellbeing in crisis line volunteers and the factors that are associated with mental wellbeing. 

## 2. Methods 

### 2.1. Search Strategy

A systematic review was conducted, with a narrative representation of the results [23]. A systematic literature search was carried out in CINAHL, Medline, PubMed, PsycINFO, and Scopus, covering the period until November 2018. Main search terms were “crisis line”, “volunteer”, and “mental wellbeing”. Comparable terms were added based on the literature found. The following terms for crisis line were used: Crisis line, crisis hotline, telephone line, telephone help line, telephone intervention, hotline, helpline, chat, chat-line, chat service, and chat support. Terms for volunteer were: Volunteer, worker, and staff. These terms were combined with terms related to mental wellbeing: mental health, professional quality of life, compassion fatigue, resilience, burnout, wellbeing, empathy fatigue, vicarious trauma, secondary trauma, secondary traumatic stress, distress, stress, help-seeking, anxiety, depressive, suicidal, supervision, treatment, service provision, skills, performance, satisfaction, exhaustion, frustration, anger, depression, and countertransference. Reference lists of the selected studies and earlier reviews [22,24] were cross-checked. 

### 2.2. Inclusion Criteria and Selection of Studies

Three inclusion criteria were used: (a) The article describes an empirical study; (b) the study samples volunteers from a crisis line or chat line; (c) the study addresses positive and/or negative impact of volunteering at the crisis line on the volunteer’s mental wellbeing. Excluded were (a) studies not written in English; (b) review studies; and (c) studies which were not published or peer reviewed.

**Figure 1:**
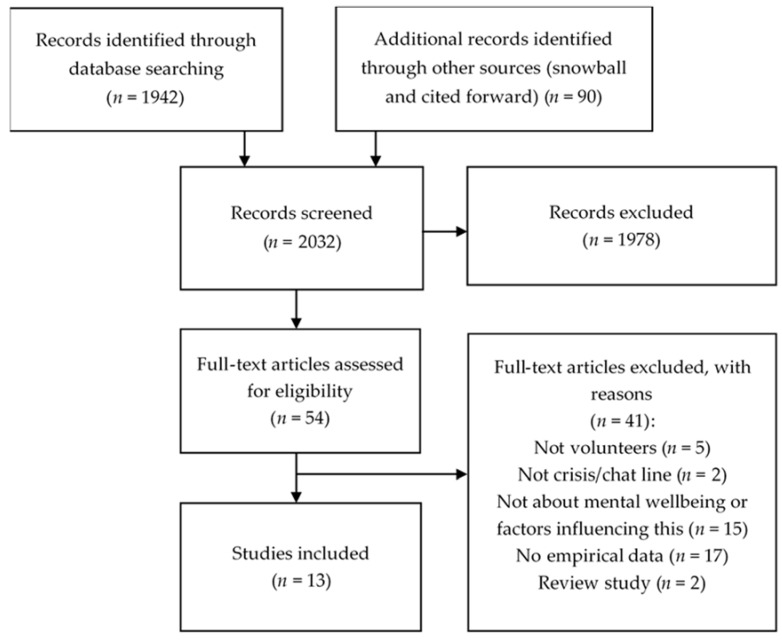
Flow chart of included studies.

The initial database search returned 1942 studies. After a first selection of useful studies, 90 additional studies were identified through reference lists and via forward citation. A total of 2032 records were screened on title and abstract by two independent reviewers (RCWJW and PV); 1978 were excluded, based upon the in- and exclusion criteria. The full texts of the remaining 54 potentially relevant articles were screened by the same reviewers. In the case of disagreements between the reviewers, a third reviewer (CHCD) was consulted. Finally, thirteen studies met the inclusion criteria and were included in the review. Figure 1 presents a flow chart of the selection process of included studies.

### 2.3. Data Extraction

Data extraction was performed by the first reviewer (RCWJW) and checked by the second (CHCD) and third (PV) reviewer. Of the thirteen included studies, the following characteristics were extracted and described in Table 1: Author(s) and publication year, sample characteristics, study design, measures, positive or negative influence on mental wellbeing, and factors associated with mental wellbeing in crisis line volunteers. Of the qualitative studies, all information, which is relevant to answer the research questions, is described in Table 1. 

### 2.4. Quality Assessment

The quality of the included quantitative (survey) studies was assessed using a 15-item quality rating list [25]. Each criteria could be scored as yes (1 point) no (0 points), or unclear (0 points). Criteria and results are presented in Appendix A Table A1. The quality of the included qualitative studies was assessed using a 9-item list of quality criteria [26], see Appendix A Table A2. Each criteria could be scored yes (1 point), no (0 points) or unclear (0 points). The scoring was conducted by two independent coders and any disagreements were discussed until consensus was reached. Due to the low number of studies found, no study was excluded based on the quality appraisal. 

### 2.5. Analysis

Since the included studies used a wide variety in outcome measures, a meta-analysis was not possible. The results will therefore be described narratively.

## 3. Results

### 3.1. Characteristics of the Included Studies

Only thirteen studies, published between 1973 and 2018, were included in the current review: eight quantitative surveys [27,28,29,30,31,32,33,34] and five qualitative studies (observation, participation, document study, and interviews) [35,36,37,38,39]. No clinical trials or longitudinal studies were found. The quality of the studies varied widely. Only four of the eight surveys used validated instruments to assess mental wellbeing or the factors influencing mental wellbeing [28,30,33,34]. The other four surveys used a single item to measure subjective mental wellbeing [27,29,31,32]. Of the qualitative studies, four presented a clear research goal or question [35,36,37,38], for example “What positive or negative experiences have volunteers had as a helpline volunteer?” [38]. The number of respondents included in the survey studies varied from 28 to 216, and in the qualitative studies from 15 to 66. The respondents in the studies were all crisis line volunteers, although two studies [28,33] also included professional employees. 

### 3.2. Negative Effects of Working at the Crisis Line on Mental Wellbeing

Ten studies investigated potentially negative effects on mental wellbeing of volunteering at the crisis line [27,28,30,31,32,33,34,36,38,39], but the outcome measures differed widely. McClure et al. [31] focused on *psychiatric diagnosis*, and found that 22% of the telephone crisis support workers met criteria for any disorder. Two studies [27,34] measured *symptoms of burnout*. Cyr and Dowrick [27] found that more than half (54%) of the volunteers in their sample ever felt burned out while working at the crisis line. Roche and Ogden [34] measured three stages of burnout (emotional exhaustion, depersonalization, personal accomplishment). A minority of the volunteers scored moderate or high on emotional exhaustion (6%); and depersonalization (15%); half of the volunteers (50%) scored low or moderate on personal accomplishment (ability to use skills). O’Sullivan and Whelan [33] found that more than three quarters of the volunteers (77%) showed symptoms of *compassion fatigue*. Dunkley and Whelan [28] reported that almost half of the respondents (46%) scored high on one of the *disruptions in beliefs* scale (safety, trust, esteem, intimacy, and control). They also reported that a quarter of the volunteers (26%) scored “quite a bit” or “extremely” on a *subjective distress* scale (hyper arousal, avoidance, or intrusion). Mishara and Giroux [32] found that pre-shift *perceived stress* in volunteers was reported as “light”. During the most stressful call perceived stress was reported “moderate”. One week after the shift perceived stress was reported as being between “light” and “moderate”. Kitchingman et al. [30] also found that more than a quarter (28%) of the volunteers scored moderate to very high on symptoms of psychological distress. In this study a few volunteers (3%) reported minimal *suicidal ideation*.

Looking at the qualitative studies, Pollock et al. [36] investigated how volunteers deal with ambiguous and anonymous conversations. They reported that *frustration and irritation* due to inappropriate calls were frequently mentioned. Yanay and Yanay [39] interviewed twenty novice volunteers and found that half of them *dropped out* immediately after training.

In sum, the studies suggest that crisis line volunteers are at risk of declined mental health. It is difficult to determine the extent of the problem, because the studies vary in the outcome measure and few used validated instruments. Consequently, prevalence rates varied widely from 3% to 77%.

### 3.3. Positive Effects of Working at the Crisis Line on Mental Wellbeing

Only five studies examined the positive effects of volunteering at the crisis line on mental wellbeing [29,33,35,37,38]. Hellman and House [29] measured *overall satisfaction*, *intent to remain* (stay volunteering at the crisis line service), and *affective commitment*, and reported that the volunteers scored on average high to very high on these variables [29]. O’Sullivan and Whelan [33] studied *posttraumatic growth* (a stable positive psychological outcome in response to a traumatic event), but found that crisis line volunteers tended to report relatively low on this measure in comparison with professional caregivers.

Four studies [32,35,37,38] gave insight into the positive effect of motivation on volunteering at the crisis line. These include both other-orientated motivations as well as self-orientated motivations*. Other-orientated motivations* mentioned are: Helping others [32], giving help the volunteer once received [32,38], feelings of altruism [35,37], and contributing to society [35]. *Self-orientated motivations* could be divided as follows (a) a purpose in life, (b) a learning experience or challenge, and (c) a new perspective on their own lives. A purpose in life includes structure in life [35], interconnectedness (meeting people, sharing experiences) [32,37], and feeling useful [32]. The learning experience was comprised of personal growth [32], deeper understanding of the human condition, and [37], developing skills and gaining experience [32,35,38]. A new perspective on their own lives includes gratefulness [35,37] and realizing their own personal blessings [37]. These motivations contributed to role-satisfaction [38] and satisfaction in general [32,35,37].

In sum, the few studies that examined the positive effects of crisis line volunteering showed that the work is satisfying and volunteers are guided by both self-orientated as well as other-orientated motivations.

### 3.4. Factors Influencing Volunteers’ Mental Wellbeing

Factors associated with mental wellbeing in crisis line volunteers were investigated by twelve of the thirteen studies. Below, we discuss the factors related to the nature of the work, factors related to the organization, and factors related to the volunteer.

#### 3.4.1. Factors Related to the Nature of the Work

Five studies mentioned factors related to the nature of the work that were negatively associated with volunteers’ mental wellbeing [27,32,36,38,39].

*Anonymity, the philosophy of non-intervention and non-disclosure*

Anonymity of the caller is an important feature of the crisis line service. For the volunteer, however, not knowing about the outcome and consequences of the contact with a caller can contribute to burnout. In addition, not having standards to evaluate success (volunteers do not know if they are doing good and if clients are improving from their help), contributed to burnout [27,36,38]. The philosophy of non-intervention may also be difficult for volunteers. This philosophy means that the volunteer only offers a listening ear and has no therapeutic function. Yanay and Yanay [39] found that the philosophy of non-intervention caused volunteers to feel confused and vulnerable and made some even consider stopping voluntary work. Pollock et al. [36] found that the principal of non-disclosure, aimed to keep focus on the caller, is inhibiting to the development of trust and confidence between callers and volunteers. Volunteers mentioned that they feel discomfort as a result of the restrictions imposed on “being oneself”.

*Urgency and length of calls*

Mishara and Giroux [32] found that higher *urgency of calls* resulted in higher levels of perceived stress during the most stressful call. Moreover, a longer *total length of calls* during a shift resulted in more perceived stress after the shift [32]. The total number of calls per shift was negatively related to posttraumatic growth [33].

*Difficult or inappropriate calls and characteristics of the callers*

Pollock et al. [36] reported in a qualitative study that volunteers experienced a lot of stress and frustration because of *(sexually) inappropriate, abusive, and manipulative calls.* It was regularly attributed as a cause of volunteers leaving the organization. Moreover, some volunteers found it difficult to deal with *complicated topics*, such as callers who are suffering from mental illness, general anxiety, unhappiness, loneliness, and social disconnectedness [36]. Sundram et al. [38] found that volunteers sometimes experienced *cultural barriers*, such as reservations about seeking help on a mental health helpline in certain callers, and volunteers needed more time than available, to clarify what callers mean when they, for example, express physical complaints instead of low mood.

#### 3.4.2. Factors Related to the Organization

Eight studies investigated the organizational factors associated with volunteers’ mental wellbeing [27,28,29,32,35,37,38,39].

*Supervision and training*

Supervision and support by a supervisor were identified as protective factors for burnout [27] and disruptions in beliefs [28]. In addition, supervision contributed to overall satisfaction, intent to remain, affective commitment [29] and job satisfaction [38]. Sufficient training was also identified as a protective factor for burnout [27] and increased job satisfaction [38]. Yanay and Yanay [39] who studied reasons for dropout, however, found that training can be very psychologically and emotionally enriching, wherefore volunteers did not start with volunteering because the training led to motivational saturation [39].

*Organizational support*

Support from the organization is an important factor for the mental wellbeing of crisis line volunteers. Frequent policy changes, a change in senior management, or rapid personnel turnover led to burnout [27]. In the study by Sundram et al. [38] several volunteers were unhappy because they felt the organization treated them as employees rather than volunteers. They felt their work was neither recognized nor appreciated [38]. Volunteers also felt underappreciated if the organization did not acknowledge their preexisting skills [38].

*Support of co-workers*

Support of co-workers is a factor that has been found to be associated with increased mental wellbeing in five studies [27,32,35,37,38]. High turnover rates of volunteers were experienced as stressful, because that hampered discussing stress and coping strategies with colleagues [27], or caused a sense of isolation [38]. The number of persons present during a shift was negatively correlated with perceived stress [32]. Three studies [32,35,37] reported that volunteers derived motivation from social connectedness, meeting other people and maintaining ties with the community.

#### 3.4.3. Factors Related to the Volunteer

Eight studies described characteristics of the crisis line volunteer that may influence their mental wellbeing [27,28,29,30,32,33,34,36]. The results are discussed below.

*Demographics and Other Specific Factors*

Younger age was found to be predictive for higher emotional exhaustion [34], general psychological distress, and the inability to manage day-to-day activities [30]. More years of experience was associated with less perceived stress [30,32]. A higher education level was found to be associated with less perceived stress [30,32]. Kitchingman et al. [30] found that women experienced more general psychological distress, but less functional impairment than men. Dunkley and Whelan [28] found that more personal trauma history led to more experience of subjective distress related to a stressful telephone call. Hellman and House [29] found that volunteers who perceived victim blaming experienced less overall satisfaction and affective commitment.

*Productive and Non-Productive Coping*

Volunteers mentioned various productive coping strategies, for example: Having realistic expectations (realizing the limits of their importance and effectiveness, and realizing that not all clients and problems will profit from help) [27]; focusing on the benefits of the voluntary work (emotional growth, education, use of helping skills, and human contact) [27]; not getting personally involved, and guarding personal boundaries [27,32,36]; “venting” with co-workers [27]; asking for feedback [27]; taking time off [27]; attending to health [27]; and relaxing activities [27].

Non-productive coping strategies were also mentioned, for example expecting appreciation from callers [27]; magical thinking (wishing that things would get better miraculously) [32]; not being able to identify and describe their own negative emotions [28,30]; not seeking help when experiencing distress [27,30]; and self-blame, worrying, and ignoring the problem [28].

More general coping styles were mentioned, such as dealing with the problem, working hard, and humor [28,29]. Finally, Roche and Ogden found a significantly positive correlation between higher emotional exhaustion and the use of an avoidant coping style [34].

*Levels of Empathy*

Two studies mentioned levels of empathy as an influencing factor on mental wellbeing [33,34]. Roche and Ogden [34] measured subscales of empathy. They found that lower levels of empathy fantasy (emotional identification with characters in books or films resulted in higher depersonalization. Lower levels of empathy concern (feeling emotional concern for others [61]) resulted in higher personal accomplishment, a subscale of burnout. O’Sullivan and Whelan [33] found that volunteers’ empathy led to more spiritual change, a subscale of posttraumatic growth. This section may be divided by subheadings. It should provide a concise and precise description of the experimental results, their interpretation as well as the experimental conclusions that can be drawn.

## 4. Discussion

Insight into the mental wellbeing of crisis line volunteers is important because a decline in mental wellbeing is associated with poorer quality and safety of care, higher absenteeism, and higher turnover rates [9]. Yet, our review showed that not much research has been conducted on wellbeing of crisis line volunteers. Despite an extensive search strategy and broad inclusion criteria, this review yielded only thirteen articles covering the period 1973–2018. Moreover, the retrieved studies varied widely in methodological quality, used a wide variety of outcome measures and most did not use validated measures.

Prevalence rates of decreased mental wellbeing ranged from 3% to 77%, showing that crisis line volunteers are at increased risk of declined mental wellbeing. It should be noted, however, that different operationalizations of decreased mental wellbeing were applied: Symptoms of burnout, compassion fatigue, vicarious traumatization, psychological disorders, distress, and feelings of frustration and irritation. To confirm a powerful conclusion about the extent of the problem, more high quality research on the wellbeing of crisis line volunteers using validated instruments is needed.

It is safe to assume that high motivation and satisfaction rates are essential for volunteer organizations, since this is after all the main reason that volunteers keep working at the organization. Only five articles studied the positive effects of volunteering. These studies demonstrated that most volunteers experience some kind of satisfaction or gratification as a result of their work at the crisis line. The qualitative studies in this review gave some insight into the motivations of crisis line volunteers. These include both other-orientated motivations as well as self-orientated motivations. Other-orientated motivations were comprised of aspects such as wanting to help others, to contribute to society, and a desire to give back. Self-orientated motivations included finding a purpose in life (to get structure, to meet other people, or to feel useful), the learning experience (personal growth, deeper understanding of the human condition, developing skills, gaining experience), and to gain a new perspective on their own lives (realizing personal blessings, being grateful). These motivations are in line with research in volunteers in general [62,63]. For example, Stukas et al. [63] found that other-oriented motives in volunteers are positively correlated with satisfaction and intentions to stay volunteering. Höing et al. [64] underlined that volunteers who are intrinsically motivated may be better protected from overburdening and burnout. More quantitative insight into how crisis line volunteering can enhance mental wellbeing is needed.

In this review we searched for factors that are associated with volunteers’ mental wellbeing. We looked for factors in three categories: (a) Factors related to the nature of the work, (b) factors related to the organization of the work, and (c) factors related to the volunteer. It should be noted that none of the studies included all these categories. Such a comprehensive approach is warranted for future research.

Factors related to the nature of the work were assessed in three studies, and include specific characteristics of the work (anonymity and philosophy of non-intervention, and non-disclosure), higher urgency of calls, inappropriate or abusive calls (i.e., repeat callers and sex callers), dealing with complex topics (i.e., loneliness, suicidality or mentally ill), and cultural barriers. Difficulties in dealing with unpredictable behavior, hostility and potential suicide patients was also found in studies among professional health care providers [14,15,16,20]. It is important that these topics are addressed in training and supervision. Several training programs have been developed for professionals to learn how to deal with a client’s inappropriate sexual and abusive behavior [65,66], but as far as we know, these have not been evaluated. It would be interesting to find out if these training programs could be a part of training for volunteers at the crisis line service. However, interventions can also be directed at the callers. For example, interventions have been developed to reduce repeat callers in crisis lines [5,6]. In addition, Baird et al. [7] and Matek [8] developed an intervention for volunteers to approach sex callers therapeutically. However, none of these interventions have been evaluated. To minimize the influence of the “difficult callers” on the mental wellbeing of crisis line volunteers, dealing with a “difficult caller” has to be an important aspect of training.

A few studies gave some insight into the factors related to the organization of the volunteer work. Supervision and training, organizational support, and support of co-workers are factors that may prevent decreased mental wellbeing. This corresponds to existing literature about volunteers in general, claiming that a positive organizational and team climate, offering acknowledgement and professional support (training, emotional support, support with daily life issues) are important determinants of mental wellbeing in trained volunteers [64]. Crisis line services should, therefore, not only pay attention to besides the quality of training and supervision, but also to the appreciation and acknowledgement of volunteers.

The third category of factors associated with mental wellbeing regards the characteristics of volunteers. Coping-mechanisms were most frequently studied. Examples of productive coping are the identification of benefits of the work, problem solving, humor, not feeling personally involved or, indicating limits to the caller. Examples of non-productive coping are magical thinking, feeling personal responsible for the outcome of the conversation, or not seeking help. Social support, realistic expectations and not feeling overly responsible for the outcome of the help are frequently studied in volunteers in general [64,67]. Crisis line services could pay attention to the realistic expectations of volunteers about the work at the crisis lines during the selection process of new volunteers. In addition, in the development of interventions attention must be paid to the cultivation of effective coping mechanisms, in order to positively influence the mental well-being of crisis line volunteers.

*Strengths and Limitations*

There are a number of strengths of this review. First, this review is an important addition to the existing literature, because it gives insight in the negative and positive impact of volunteering at the crisis line. Second, this is the first review that gives an overview of the factors associated with mental wellbeing in crisis line volunteers. Finally, by using a broad search string and inclusion criteria, we were able to find more studies than the previous systematic review on the topic [22] and we believe that we have included all relevant published articles.

There are several limitations to this review that must be mentioned. Firstly, our search yielded two relevant dissertations about this topic, which were not published in peer reviewed journals [68,69]. Despite multiple efforts to contact the authors or retrieving the dissertations via university libraries, we did not succeed in obtaining the dissertations. As a result, relevant information could have been missed. Secondly, there might be a risk of publication bias. Thirdly, five of the thirteen studies scored low on the quality assessment (≤67% of criteria are sufficient). Although the limited quality of these studies may limit the results of our review, we decided to include all studies, because only very few studies were found and therefore all studies have an added value for this review.

## 5. Conclusions

Remarkably few studies have examined mental wellbeing in crisis line volunteers. These studies suggest that volunteers are at risk of decreased mental wellbeing, despite the gratification they experience from their work. More high quality research with validated instruments is needed to get a better view of the prevalence of decreased mental wellbeing. Our results show that a variety of work-related, organization-related, and volunteer-related factors seem to be associated with a mental wellbeing of crisis line volunteers. However, more comprehensive research, studying all these factors, is necessary. In addition, there is a need for interventions targeting these factors, to ensure the high quality of care and to maintain or increase the mental wellbeing of crisis line volunteers.

## Figures and Tables

**Table 1:** Characteristics of included studies.

Author(s) and Date	Design and Sample Characteristics	Measures	Results on Positive and Negative Mental Wellbeing	Factors Influencing Mental Wellbeing
Cyr and Dowrick (1991) [27]	Design: Survey, descriptive. Respondents (*n* = 39) Female: 69% Age: 74% under age of 40 Active volunteers: 62% Mean years of experience at the crisis line: 52% more than a year Mean hours per month: 16 h	Mental wellbeing measure: Burnout Questionnaire (developed for this study, 1 item): “Have you ever felt burned-out from working on the crisis line” Checklist of burnout stages: (a) Excessive Enthusiasm (great expectations and excessive energy in volunteer work), (b) Stagnation (disappointment from lack of fulfilment of initial expectations), (c) Frustration (doubts of effectiveness and the value), and (d) Apathy (an emotional detachment, a feeling of numbness or boredom along with an attitude of resignation – mechanically going about duties, the loss of feeling care/concern for clients). Factors related to mental wellbeing: Self-reported factors for preventing/managing burnout and factors contributing to burnout.	54% of the respondents have felt burned out on the crisis line. 75% indicated that the feeling of burnout arose within a year. 97% of all respondents experienced at least one stage of burnout within one year: Excessive enthusiasm: 77%; stagnation: 18%; frustration: 39%; apathy: 28%.	Factors related to the nature of work: Lack of completion in the volunteer work (28%) (anonymity) Factors related to the organization: Factors managing/preventing burnout: Supervisor support (67%); Feeling of being appreciated (67%); Sufficiently trained to perform as volunteer work (62%); Attendance of a reasonable number of volunteer meetings was required (56%). Factors contributing to burnout: Volunteer turnover (44%); Lack of contact with volunteers (36%); Lack of discussion about work stresses and/or complaints among peer volunteers (36%); Lack of identification by the volunteer group of ways to manage burnout (31%). Factors related to the volunteer: Factors managing/preventing burnout: Identification of some benefits from the volunteer work (69%); Realizing the limits of your importance and effectiveness (59%); Realizing that clients cannot or do not always make the most of help available (56%); Realizing that not all clients and problems will profit from help (56%). Factors contributing to burnout: No standards to evaluate success (31%); Feeling of incompetence in counselling skills (31%); Expecting appreciation (28%); Lack of completion in the volunteer work (28%). Strategies for managing burnout: Setting limits on involvement (e.g., limiting volunteer hours) Avoiding high expectations “Venting” with peer volunteers and staff (e.g., debriefing, expressing feelings) Requesting performance evaluations Taking time off Attending to health Engaging in relaxing activities Nonproductive coping: Not seeking help from supervisor
Roche and Ogden (2017) [34]	Design: Survey, descriptive. Respondents (*n* = 216) Female: 69.1% Age: (*M* = 28.7, *SD* = 11.81), Range 18–80. Active volunteers: 100% Mean years of experience at the crisis line: no information Mean hours per week: (*M* = 4.17, *SD* = 1.7), Range 1–15 h.	Mental wellbeing measure: Burnout: using the Maslach Burnout Inventory (MBI-HSS; Maslach, 1982) modified for Samaritans’ listening volunteers. This 22 item questionnaire has three subscales: (a) Emotional Exhaustion; low: 0–16, moderate: 17–26, high: 27 or over. (b) Depersonalization; low: 0–6, moderate: 7–12, high: 13 or over. (c) Personal Accomplishment; low: 39 or over; moderate: 32–38; high: 0–31 [40]. Factors related to mental wellbeing: Social Support: using the Short Form Social Support Questionnaire (SSQ6; Sarason, Shearin, Pierce, and Sarason, 1987) [41]. Empathy: using the Interpersonal Reactivity Index (IRI; Davis, 1983); Perspective Taking, Fantasy, Empathic Concern and Personal Distress [42]; Coping: using the Brief COPE (Carver, 1997) grouped into two subscales; approach and avoidant [43].	Emotional exhaustion: Low = 203 (94%) 0–16 Moderate = 13 (6%) 17–26 High = 0 (0%) 27 or over Depersonalization: Low = 184 (85.2%) 0–6 Moderate = 27 (12.5%) 7–12 High = 5 (2.3%) 13 or over Personal accomplishment: Low = 55 (25.5%) 39 or over Moderate = 52 (24.1%) 32–38 High = 109 (50.5%) 0–31	Factors related to the volunteer: *Demographic variables:* Gender, living arrange and diary keeping were not significantly related to the burnout scales. Age: Younger age predicted higher emotional exhaustion accounting for 9.7% of the variance (*F* = 2.9; *p* = 0.001). There was no significant relation with depersonalization and personal accomplishment. *Empathy:* The perspective taking and empathic concern empathy scales were not significant related to the burnout scales. Lower empathy fantasy predicted greater depersonalization scores accounting for 12.7% of the variance (*F* = 3.6; *p* = 0.0001). There was no significant relation with emotional exhaustion and personal accomplishment. Lower empathy concern predicted higher personal accomplishment accounting for 6.3% of the variance (*F* = 2.2; *p* = 0.01). There was no significant relation with emotional exhaustion and depersonalization. *Coping:* Approach coping was not significant related to the burnout scales. Use of an avoidant coping style predicted higher emotional exhaustion accounting for 9.7% of the variance (*F* = 2.9; *p* = 0.001). Greater avoidant coping predicted greater depersonalization scores accounting for 12.7% of the variance (*F* = 3.6; *p* = 0.0001). Avoidant coping was not significant related to personal accomplishment. Non-significant values are not included in the table.
Dunkley and Whelan (2006) [28]	Design: Survey, correlational. Respondents (*n* = 64) Female: 88.7% Age: (*M* = 45.54, *SD* = 11.59), range: 24.7–75.2 years. Active volunteers: 49.9% volunteers, 58.1% paid counsellors, Mean years of experience at the crisis line: 3,4 years (*SD* = 3.31 years).	Mental wellbeing measure: TABS (Trauma Attachment and Belief Scale) [44]. Assesses disruptions in beliefs, related to five need areas that are sensitive to the effects of trauma: Safety, Trust, Esteem, Intimacy and Control. 84 items, 5-point Likert scale. Possible range 20–80. Average: 45–55; high average: 56–59; very high: 60–69; extreme high: >70. IES-R (Impact of Event Scale – Revised) [45]. Assesses subjective distress over past 7 days related to any specific life event. Developed to parallel three of the four PTSD criteria: Hyperarousal, Avoidance and Intrusion. 22 items, 4-point Likert scale (0–4). No cut-off points. Factors related to mental wellbeing: CSA (The Coping Scale for Adults) [46]. Assesses four coping styles: Dealing with the problem, Sharing, Optimism, and Non-productive coping. High scores indicate that participants are using a coping style frequently. 20 items, on a 5-point Likert scale (1–5). SWAI (The Supervisee From the Supervisory Working Alliance Inventory) [47]. Assesses the supervisee’s perceived working alliance with their supervisor. 19 items on a 7-point Likert scale (1–7). Trauma history (no description of the construct)	Mean total score on the TABS (*M* = 45.28, *SD* = 8.18) was in the average range), 4.8% had total scores in the high average range and 3.2% scored in the very high range. 45.9% respondents had high average to extreme high scores on at least one of the TABS subscales). Mean total score for the IES-R was low, given the possible range of 0–84 (*M* = 9.21, *SD* = 10.36). 25,9% respondents answered “quite a bit” or 4 “extremely” on at least one IES-R question.	Factors related to the organization: Standard multiple regression of predictors and Total Trauma Attachment and Belief Scale total scores (TABS): *R²* (25% adjusted) was significantly different from zero, *F*(6, 51) = 4.10, *p* = 0.00, with 33% of the variance explained. ○ Supervision (whether participants received supervision), b * = 0.14, *t* = 1.12, *p* = 0.27 Standard multiple regression of predictors and Total Impact of Event Scale-Revised scores (IES-R): regression analysis was not significantly different from zero, *F*(6, 48) = 1.47, *p* = 0.21. Supervisee total score was negatively correlated with the TABS total score (*r* = −0.36, *p* = 0.02) Supervisee total score was not significant correlated with the IES-R total score (*r* = −0.26, *p* = 0.10). Factors related to the volunteer: Standard multiple regression of predictors and Total Trauma Attachment and Belief Scale total scores (TABS): *R*² (25% adjusted) was significantly different from zero, *F*(6, 51) = 4.10, *p* = 0.00, with 33% of the variance explained. Non-productive coping, *b* * = 0.52, *t* = 3.66, *p* = 0.00 Dealing with the problem, *b* * = −0.35, *t* = −2.41, *p* = 0.02 Optimism, *b* * = −0.19, *t* = −1.19, *p* = 0.24 Sharing, *b* * = 0.10, *t* = 0.71, *p* = 0.48 Personal trauma history, b* = −0.00, *t* = −0.03, *p* = 0.98 Standard multiple regression of predictors and Total Impact of Event Scale-Revised scores (IES-R): regression analysis was not significantly different from zero, *F*(6, 48) = 1.47, *p* = 0.21. Non-productive coping was positively correlated with the TABS total score (*r* = 0.38, *p* < 0.01), Dealing with the problem was negatively correlated with the TABS total score (*r* = −0.31, *p* < 0.05), Optimism (*r* = −0.11) and sharing (*r* = −0.19) were not significant correlated with the TABS total score (*p* > 0.05); Non-productive coping (*r* = 0.23), dealing with the problem (*r* = 0.04), optimism (*r* = 0.14) and sharing (*r* = −0.04) were not significant correlated with the IES-R total score (*p* > 0.05); Personal trauma history was not significant negatively correlated with the TABS total (*r* = −0.08, *p* > 0.05); Personal trauma history was significant positively correlated with the IES-R total score (*r* = 0.28, *p* < 0.05).
Kitchingman et al. (2016) [30]	Design: Survey, correlational. Respondents (*n* = 210). Female: 78.1%. Age: (*M* = 49.05, *SD* = 14.23), range: 20–75 years [48]. Active volunteers: 100%. Mean years of experience at the crisis line: 0–2 years.	Mental wellbeing measure: K10 [49], assesses general psychological distress. 10 item measure, 5-point Likert scale (1–5). Possible range 0–50. Low: 0–15; moderate: 16–21; high: 22–29; very high: 30–50. ASIQ-CI (suicidal ideation) [50], assesses the intensity and lethality, together with specificity and availability of a suicide plan in the last month. 7 item measure, 7-point Likert scale. Minimal: 0–8; moderate: 9–32; critical: 33–48. Functional impairment (two additional items from the K10 [49]): “days out of role” (DOR) and “days cut back” (DCB). Possible range 0–28. Factors related to mental wellbeing: Demographics: Categorical items were used to assess participants’ age, sex, location (regional/rural/remote, metropolitan), highest educational qualification (university degree, none/high school/apprenticeship/diploma), and number of years of experience as a TCS. TAS-20 (Toronto Alexithymia Scale) [51], assesses difficulty identifying and describing feelings. Three subscales: Difficulty Identifying Feelings; Difficulty Describing Feelings; and Externally Oriented thinking. 20 items on a 5-point Likert scale (1–5). The externally oriented thinking subscale was excluded (8 items). Possible range 11–55. GHSQ-V (General Help-Seeking Questionnaire – Vignette version)) [52], assesses help-seeking using intention, two subscales: Intentions to seek help for psychological distress; and Intentions to seek help for suicidal ideation. Both subscales have 3 items on a one 7-point Likert scale (1–7).	General psychological distress (*M* = 14.10, *SD* = 4.35). 71,9% reported low symptoms, 28.1% reported moderate to very high symptoms of psychological distress. Suicidal ideation (*M* = 2.00, *SD* = 3.30). 97.1% reported minimal suicidal ideation, 2.9% reported moderate suicidal ideation. “Days out of role” (*M* = 0.23, *SD* = 1.84) and days cut back (*M* = 1.17, *SD* = 4.43)	Factors related to the volunteer: Respondents who reported moderate to high symptoms of psychological distress, reported lower intention to seek help *F*(1, 204) = 4.09, *p* = 0.044. Respondents who reported moderate suicidal ideation also reported lower intentions to seek help *F*(1, 204) = 8.76, *p* = 0.003; Difficultly identifying and describing feelings was significantly correlated with: general psychological distress (*r* = 0.53), suicidal ideation (*r* = 0.25), intentions to seek help for psychological distress (*r* = −0.15), intentions to seek help for suicidal ideation (*r* = −0.14), had to cut down day-to-day activities (*r* = 0.19); Difficultly identifying and describing feelings was not significant correlated with being totally unable to manage day-to-day activities (*r* = −0.01). Age was significant correlated with general psychological distress (*r* = −0.30) and being totally unable to manage day-to-day activities (*r* = −0.17) Gender was significant correlated with: intentions to seek help for psychological distress (r = 0.15), being totally unable to manage day-to-day activities (r = −0.15), and having to cut down on day-to-day activities (r = −0.15) Education was significant related to difficulty in identifying and describing feelings (*r* = −0.15). Years of experience at the crisis line was significant correlated with having to cut down day-to-day activities (*r* = 0.17)
McClure et al. (1973) [31]	Design: Interviews conducted by researcher and two psychiatrists Self-selecting. RespondentsCall For Help (CFH) and (*n* = 74) Youth Life Line (*n* = 51). Total (*n* = 125). Active volunteers: 100%	Mental wellbeing measure: Psychiatric diagnosis by interviewer and two psychiatrists (diagnostic criteria unspecified)	Current illness CFH: Depressive disorder: 7% Antisocial personality: 3% Possible psychosis: 3% Other neurosis: 3% No psychiatric disorder: 78% Current illness YLL: Depressive disorder: 4% No psychiatric disorder: 96%	
Mishara and Giroux (1993) [32]	Design: Survey, correlational. Respondents (*n* = 80) Female: 51% Age: (*M* = 28.6 years, *SD* = 9.94), range 19–64 years. Active volunteers: 100%. Mean years of experience at the crisis line: 9.1 months (*SD* = 12.7 months),	Mental wellbeing measure: Level of perceived stress concerning their telephone intervention work before they started the shift, after the shift (perceived stress during the most stressful call), and after the shift when they were contacted later (level of stress concerning their previous telephone intervention shift). One item, on a visual analogue scale from 0–100. No description of cut-off points Factors related to mental wellbeing: Ways of Coping Checklist, revised version [53], assesses possible cognitive and behavioral coping strategies. 66 items on a 4-point Likert scale; Personal experiences with suicide [54,55]; Motivations for doing volunteer work with suicidal people, open question; Certain beliefs concerning their role as volunteer, open question.	Perceived stress before shift was called “light” (*M* = 29.80, *SD* = 19.14) during the most stressful call “moderate” (*M* = 49.74, *SD* = 21.47), after shift between “light” and “moderate” (*M* = 33.85, *SD* = 22.90).	Factors related to the caller/nature of the problem/care question: Urgency of call (*F* = 27.86, *p* < 0.001), with 26.8% of the variance explained, total length of calls (*F* = 6.13, *p* < 0.05), with 32.4% of the variance explained, were positively correlated with perceived stress during the most stressful call; Total length of calls (*F* = 24.29, *p* < 0.001), with 24.2% of the variance explained, was positively correlated with perceived stress after the shift. Factors related to the organization: Number of persons present during shift, is negatively correlated with perceived stress after the shift (*F* = 9.69, *p* < 0.01), with 32.9% of the variance explained. Factors related to the volunteer: Experience at the crisis line was negatively correlated with perceived stress before shift (*F* = 11.15, *p* < 0.001), with 12.8% of the variance explained; Magical thinking (*F* = 4.70, *p* < 0.05), with 36.4% of the variance explained and feeling personally responsible (*F* = 3.99, *p* < 0.05), with 43.2% of the variance explained, were positively correlated with perceived stress during the most stressful call. Detachment (*F* = 4.51, *p* < 0.05), with 40.1% of the variance explained, was negatively correlated with perceived stress during the most stressful call; Magical thinking (*F* = 5.01, *p* < 0.05), with 46.3% of the variance explained, was positively correlated with perceived stress after the shift. Education (*F* = 6.28, *p* < 0.01), with 38.1% of the variance explained. Realistic expectations (*F* = 5.55, *p* < 0.05), with 42.5% of the variance explained, and positive thinking (*F* = 4.39, *p* < 0.05) with 49.4% of the variance explained, were negatively correlated with perceived stress after the shift. Factors related to volunteers’ motivation: To help others 98%, to gain experience 88%, for personal growth 90%, to meet people 70%, to share my experience 53%, to feel useful 76%, to give help I once received 43%.
Pollock et al. (2012) [36]	Design: Qualitative, by observation of volunteers’ activities and interviews. Respondents (*n* = 66) Active volunteers: 100%.	Three central themes are described: (a) How volunteers categorized calls and configured the caller in intrinsically ambiguous and anonymous encounters; (b) Volunteer strategies of self-protection from abusive and manipulative calls; and (c) How these strategies of categorization and self- protection resulted in the judging of calls and callers. Information about the last category is not mentioned, because this information is beyond the scope of this study.	Frustration and irritation over the bad/inappropriate calls was a frequent topic in interviews and in discussion with and between branch volunteers. It was regularly attributed as a cause of volunteers leaving the organization.	Factors related to the nature of the work: Topic of the call: (sexually) inappropriate, abusive, and manipulative calls. Callers who are suffering from mental illness, general anxiety, unhappiness, loneliness and social disconnectedness. The principal of non-disclosure, aimed to keep focused on the caller. It inhibits the development of the trust and confidence between callers and volunteers. Factors related to the volunteers: Doubt and uncertainty due to “good” or “genuine” contact. Insufficient resources to handle abusive and violent calls. Insufficient access to in-call strategies for distancing and self-protection. Strategies of self-protection: indicate limits directly to callers and guard personal boundaries, refocus inappropriate calls to the reason for calling the crisis line and the emotion of the caller.
Sundram et al. (2018) [38]	Design: Qualitative (focus groups and in-depth interviews). Respondents (*n* = 25) Female: 99.5% Age: 25–67 years. Mean experience: 3 years (range: 1–15 years).	Three key questions that are relevant to this review were answered: (a) What are the key motivations for starting volunteering? (b) What positive or negative experiences have volunteers had as a helpline volunteer?; (c) What factors are associated with volunteers’ job satisfaction and intention to stay?; (d) What factors are associated with an intention to leave?	Extrinsic motivation to start volunteering is to give back to the wider community what others had or had not been able to give to them. The intrinsic motivation to start volunteering was to gain skills and work experience and to develop new skills. Making a difference, helping the caller and phone calls ending on a positive note.	Factors related to the nature of the work: There were cultural barriers such as stigma in certain callers. More time is necessary to clarify what the callers’ needs were as they were sometimes framed as physical complaints instead of low mood. Factors related to the organization: A supportive network enabled by the organization during supervision leads to job satisfaction. Social support of other volunteers. The high quality of the new volunteer training program and ongoing supervision leads to job satisfaction. Development of a range of skills in training was not only focused on counselling, but also on self-growth and self-care. The organization did not explore the skill set of volunteers. This lead to feelings of being underappreciated. Volunteers felt underappreciated which affected the volunteers’ sense of belonging with the organization. Inconsistent communication about changes in the organization. A high turnover and differences in motivation of student volunteers leads to dissatisfaction and a sense of isolation in the long-term volunteer. New technology changes could be more user friendly.
Yanay and Yanay (2008) [39]	Design: Qualitative study by observation, participation, document study and interviews. Respondents (*n* = 20) Female: 100% Active volunteers: volunteers who dropped out after training and volunteers who dropped out after a year.	The observation, participation and document study resulted in a description of the content and the atmosphere of the training. The interview question was: “Tell me everything that happened to you from the moment you decided you wanted to volunteer until the day you dropped out”. This study is looking at volunteerism through the phenomenon of dropping out.	Feelings of secondary trauma led to dropout within a year. Dropout rate was very high immediately following the course (about 50 percent). The percentage of dropouts among those who had not begun work on the hotline was higher than among volunteers who did begin working and left (about 25 percent). Dropout numbers among young volunteers were higher than among older volunteers, women who had previous volunteering experience persevered longer than those who had never volunteered before, volunteers who were victims of sexual violence stayed longer.	Results are abstracts from observations and interviews. Factors related to the organization: Volunteers experienced the training as very powerful and fulfilling emotionally, socially, and intellectually, and that the course had a dramatic impact on their consciousness, knowledge, and interrelations. The course sparked great ambivalence and conflict. Volunteers did not start with volunteering because the training led to motivational saturation. Voluntary organizations often hold the view that volunteerism is based on free will and choice, and that they therefore should not be prompting or motivating volunteers. This approach, however, conveyed to the volunteers that perhaps they were not really needed by the organization. The organizational philosophy of freedom and non-intervention that perceives volunteers as autonomous agents remained tacit and misunderstood. It can give rise to anger and feelings of abandonment, eventually leading to volunteers’ dropout. Factors related to the volunteer: Lack of knowledge on how to manage emotional difficulties and work ambiguity led to feelings of confusion, overload and a growing feeling of vulnerability. This was the leading reason for dropout after a year.
O’Sullivan and Whelan (2011) [33]	Design: Survey, correlational. Respondents: (*n* = 64) Female: 70.3% Age: (*M* = 44.84, *SD* = 15.16), range 18–72 years Active volunteers: 76.6% volunteers, 23.4% paid counsellors Mean experience: 3.24 years (*SD* = 46.68 months).	Mental wellbeing measure: PTGI (Post Traumatic Growth Inventory) [56], assesses positive outcomes in relation to either a recent or salient traumatic event (in this study telephone call). Five subscales: Relating to others, New possibilities, Personal strength, Spiritual change and Appreciation of life. 21 items on a 5-point Likert scale (0–5), possible range 0–105. ProQol (Professional Quality of Life) [57], measure professional quality of life in three scales: Compassion Satisfaction, Burnout and Compassion Fatigue. Only scores from the Compassion Fatigue scale were used for analysis. 10 items on a 5-point Likert scale (0–5) for Compassion Fatigue, possible range: 0–50. Scores below 8 are considered as “not concerning”, 8–17 are “concerning” and above 18 may suggest “something about work is frightening” [58]. Factors related to mental wellbeing: JSPE (Jefferson Scale of Physician Empathy) [59]: measures empathy in a professional helping context. 20 items on a 5-points Likert scale. CSS (Crisis Support Scale) [60]: measures received support following a crisis. 7 items on a 7-point Likert scale. Calls per shift	Posttraumatic growth (*M* = 41.34, *SD* = 21.00). Compassion fatigue: 43% scored less than 8, 60,9% scored between 8–17 and 17.2% scored above 17. Compassion fatigue was significant positive correlated with Posttraumatic Growth (*r* = 0.26, *p* < 0.05), specifically in the subscale “Relating to Others (*r* = 0.26, *p* < 0.05) and Personal Strength (*r* = 0.35, *p* < 0.05). Compassion Fatigue was predicted by posttraumatic growth *F*(6, 57) = 2.38, *p* < 0.05, with 16% of the variance explained.	Factors related to the organization: Crisis support was not significant correlated to posttraumatic growth (*r* = −0.09, *p*> 0.05) or compassion fatigue (*r* = −0.16, *p* = > 0.05); Calls per shift was negatively related with the subscale of posttraumatic growth “relating to others” *F*(6, 57) = 2.38, *p* < 0.05. Factors related to the volunteers: Empathy was not significant related to compassion fatigue (*r* = −0.001, *p* ≥ 0.05); Empathy was not significant related to overall posttraumatic growth (*r* = 0.17, *p* ≥ 0.05), but positive related to the subscale of posttraumatic growth “spiritual change” (*r* = 0.30, *p* < 0.05).
Hector and Aguirre (2009) [35]	Design: Qualitative. Respondents (*n* = 15) Female: 75% Age: Between 24 and 66+ years old Active volunteers: 100%. Mean years of experience at the crisis line: 9. Respondents had volunteered for over five years	The motivation volunteers get from their work.	All respondents indicated that they are motivated to work at the crisis line.	Factors related to volunteers’ motivation: Feelings of contributing to society, feelings of altruism, challenging, informative, grateful, structure to life.
Hellman and House (2006) [29]	Design: Survey, correlational. Respondents: (*n* = 28). Active volunteers: 100%.	Mental wellbeing measure: Overall satisfaction, single item measure: “Overall, I am satisfied with my experience as a volunteer with (name centre)”. 5-point Likert scale (1–5). Intent to remain, single item measure: “Over the next year, how likely are you to continue as a volunteer for (name centre)” 5-point Likert scale (1–5). Affective commitment: assesses the emotional attachment that the participant has with the specific organization. Five-item measure, 5-point Likert scale (1–5). Possible range 5–25. Factors related to mental wellbeing: ACS (emotional attachment with the organization) Perceived value monthly meetings Crisis volunteer self-efficacy Social support Perceived experience with victim blaming Questionnaires were developed for this study	Overall satisfaction (*M* = 4.6, *SD* = 0.6), Possible range: 1–5. Intent to remain (*M* = 4.7, *SD* = 0.5), possible range: 1–5. Affective commitment (*M* = 19.7, *SD* = 3.6), possible range: 5–25.	Factors related to the organization: Perceived value of monthly meetings was significant positively correlated with overall satisfaction (*r* = 0.55, *p* = 0.003), intent to remain (*r* = 0.50, *p* = 0.008) and affective commitment (*r* = 0.34, *p* = 0.083). Factors related to the volunteer Self-efficacy was significant positively correlated with overall satisfaction (*r* = 44, *p* = 0.019) and affective commitment (*r* = 0.50, *p* = 0.007), but not significant with intent to remain (*r* = 0.21, *p* = 0.28); Social support was significant positively correlated with overall satisfaction (*r* = 0.49, *p* = 0.012), but not significant with intent to remain (*r* = 0.19, *p* = 0.34) or affective commitment (*r* = 0.14, *p* = 0.488); Perceived experience with victim blaming was significant negatively correlated with overall satisfaction (*r* = −43, *p* = 0.021) and affective commitment (*r* = −0.36, *p* = 0.058), but not significant with intent to remain (*r* = 0.19, *p* = 0.34).
Praetorius (2005) [37]	Design: Qualitative. Respondents (*n* = 19) Female (*n* = 17). Age: 18–66 years old. Active volunteers: 100%. Mean years of experience at the crisis line: from less than a year to over 16 years	Benefits and motivation of volunteering at the hotline and reasons for coming back	Volunteers are coming back to the crisis hotline.	Factors related to volunteers’ motivation: Altruism (desire to give back), realizing personal blessings (gaining a new perspective of one’s own life, perceived challenges and obstacles), a deeper understanding of the human condition, interconnectedness among us all as part of the social fabric.

## Appendix A

**Table A1:** Quality assessment for all included survey studies, in order of year of publication.

Criterion *																
	Objec-tive	Design	Target Population and Sample							Varia-Bles	Data Sources/Collection	Measure-Ment		Statistics		Score out of 15
	1	2	3a	3b	3c	3d	3e	3f	3g	4	5a	5b	5c	6a	6b	
McClure et al. (1973)	Y	N	N	N	N	N	N	N	Y	Y	Y	N	N	Y	Y	6
Cyr & Dowrick (1991)	Y	Y	Y	N	Y	Y	N	Y	N	Y	Y	N	Y	N	N	9
Mishara & Giroux (1993)	Y	Y	Y	N	N	Y	Y	Y	N	Y	Y	N	N	Y	N	9
Hellman & House (2006)	Y	Y	Y	N	Y	Y	N	N	Y	Y	Y	Y	N	Y	Y	11
Dunkley & Whelan (2006)	Y	N	Y	N	Y	Y	Y	Y	Y	Y	Y	Y	Y	Y	Y	13
O’Sullivan & Whelan (2011)	Y	N	Y	N	N	Y	N	Y	Y	Y	Y	Y	Y	Y	Y	11
Kitchingman et al. (2016)	Y	Y	Y	Y	N	Y	Y	Y	Y	Y	Y	Y	Y	Y	Y	14
Roche & Ogden (2017)	Y	Y	N	Y	N	N	N	Y	Y	Y	Y	Y	Y	Y	N	10

* (1) Are the objectives or hypotheses of the research described in the paper stated?; (2) Is the study design presented? (3a) Do the authors describe the target population they wanted to research? (3b) Was a random sample of the target population taken? AND was the response rate 60% or more? (3c) Is participant selection described? (3d) Is participant recruitment described, or referred to? (3e) Are the inclusion and/or exclusion criteria stated? (3f) Is the study sample described? (minimum description sample size, gender, age) (3g) Are the numbers of participants at each stage of the study reported? (Authors should report at least numbers eligible, numbers recruited, numbers with data at baseline, and numbers lost to follow-up). (4) Are the measures and outcome described? (5a) Do authors describe the source of their data AND did authors describe how the data were collected? (e.g., by mail) (5b) Was reliability of the measure(s) mentioned or referred to? (5c) Was the validity of the measure(s) mentioned or referred to? (6a) Were appropriate statistical methods used and described, including those for addressing confounders? (6b) Were the numbers/percentages of participants with missing data for sitting and the health outcome indicated AND If more than 20% of data in the primary analyses were missing, were methods used to address missing data?

**Table A2:** Quality assessment for all included qualitative studies, in order of year of publication (*n* = 5).

	Criterion									Total out of 9
	Clear Question?	Theoretical Framework and Methods Explicitly Defined?	Selection Clearly Described and Theoretically Complete?	Fieldwork Described in Detail?	View Raw Data and Transcription by Others?	Analysis Clearly Described and Theoretically Substantiated?	Analysis by More Than One Researcher?	Explicitly Searched for Counter-Examples?	Display of Convincing Empirical Material?	
Praetorius & Machtmes, (2005)	Y	Y	Y	Y	N	Y	Y	N	N	6
Hector & Aguirre, 2008	Y	Y	Y	Y	N	Y	Y	Y	Y	8
Yanay & Yanay, 2008	N	Y	Y	Y	N	N	Y	Y	Y	6
Pollock, et al., 2012	Y	Y	Y	Y	Y	Y	Y	Y	Y	9
Sundram et al. (2018)	Y	Y	Y	Y	Y	Y	Y	Y	Y	9
